# Regenerative medicine funding policies in Europe and The Netherlands

**DOI:** 10.1038/s41536-016-0006-8

**Published:** 2017-01-05

**Authors:** Gerald de Haan, Rini de Crom, Elaine Dzierzak, Christine Mummery

**Affiliations:** 1 Scientific Director European Research Institute for the Biology of Ageing, University Medical Center Groningen, University of Groningen, Groningen, The Netherlands; 2 Director of Biomedical Sciences, Erasmus MC, Rotterdam, The Netherlands; 3 Professor of Haematological Regeneration, MRC Centre for Inflammation Research, University of Edinburgh, Edinburgh, UK; 4 Chair Department of Anatomy and Embryology, Leiden University Medical Center, Leiden, The Netherlands

Over the last decade, stem cell biology has undergone a revolution in technological advances that will collectively have major and long-lasting impact on regenerative medicine. These include the ability to generate pluripotent stem cells from adult bodycells and to grow mini-organs from these or from adult stem cells in defined culture conditions. Both approaches provide ways to derive functional cells of human tissue that could be used for transplantation and tissue repair. Laboratories worldwide now produce cardiomyocytes, blood cells, insulin-producing B-cells, liver, and even brain cells entirely routinely. A major advantage of these approaches is that they enable the use of a patient’s own body material to grow new cells and tissues, thereby preventing life-long treatment with drugs to avoid rejection. The future of stem cell biology and regenerative medicine looks extremely bright. At the same time, however, we have witnessed a number of incidents of dubious or even fraudulent clinical interventions based on stem cells. The promise of stem cells and regenerative medicine to benefit patients is huge but has led to opportunistic and disproportionate incentives to push results from basic science into the clinic.

It is our firm conviction that acquiring knowledge and developing technologies to use stem cells for clinical application is best carried out in networks or virtual institutes in which scientists from multiple disciplines collaborate, critically assess each others’ results and create synergy by bringing together knowledge and expertise from different subdisciplines. We have participated for many years in multiple of these networks that were funded by the European Commission or by the Dutch Government, including EuroStemCell and EuroSystems (http://www.eurostemcell.org), and the Netherlands Institute for Regenerative Medicine (http://www.nirmresearch.nl). In these networks, the best scientists in the various subdisciplines of regenerative medicine were united, training a new generation of young researchers and medical doctors, and organizing courses and summer schools to ensure interaction, communication of the newest developments, and providing education in specific skills, like managing intellectual property and entrepreneurship. In addition, they organized public outreach activities. In spite of positive evaluation by external advisory boards, these networks are essentially one-time events, with no options for (competitive) renewals. Thus, under the current regulations, collaborative networks inevitably come to an end, no matter how successful they have been.

The European Union (EU) and Dutch government have prioritized regenerative medicine as an area of key strategic relevance, but networks of stem cell biologists can only apply in response to requests for applications, or ‘calls’. These call texts are often very specific and limited in scope, and how they are defined is less than transparent for most researchers. In the current EU research program, Horizon 2020, stem cell calls are all worded to expedite clinical use. While no one in the field denies the wide clinical potential of stem cells, a single focus on clinical use without a fundamental research base is bound to disappoint public, patients, and politicians alike.

The Dutch government has embarked on a process to solicit and then shortlist research questions originating from the public, leading to the “Dutch National Research Agenda” (http://www.wetenschapsagenda.nl/?lang=en). Regenerative Medicine is now confirmed as a publicly approved topic of national interest. What is needed is a transparent funding scheme that will promote the combination of insights from stem cell biologists, scientists working on applied regenerative medicine, specialists in materials science and others to develop a pipeline that will bring new fundamental insights into the clinic in a sustainable way. The long-term establishment of multi-disciplinary networks is the only sound way to achieve this.

